# Peer Review of “Use of Spinal Anesthesia in Pediatric Laparoscopic Appendectomies: Case Series”

**DOI:** 10.2196/29607

**Published:** 2021-04-28

**Authors:** Theodoros Aslanidis

**Affiliations:** 1 Intensive Care Unit St Paul General Hospital Thessaloniki Greece

**Keywords:** pediatrics, appendectomy, spinal anesthesia, general anesthesia, laparoscopy, vomiting, keyhole, surgery, anesthesia, appendix

*This is a peer-review report submitted for the paper “Use of Spinal Anesthesia in Pediatric Laparoscopic Appendectomies: Case Series”.*

## Round 1 Review

### General Comments

A good written presentation of the study [1] was conducted by the authors.
